# Nudibranchs as Sources of Marine Natural Products with Antitumor Activity: A Comprehensive Review

**DOI:** 10.3390/md23080319

**Published:** 2025-08-03

**Authors:** Máximo Servillera, Mercedes Peña, Laura Cabeza, Héctor J. Pula, Jose Prados, Consolación Melguizo

**Affiliations:** 1 Institute of Biopathology and Regenerative Medicine (IBIMER), Center of Biomedical Research (CIBM), University of Granada, 18100 Granada, Spain; servillera03@correo.ugr.es (M.S.); mpenacontreras@ugr.es (M.P.); lautea@ugr.es (L.C.); melguizo@ugr.es (C.M.); 2 Instituto de Investigación Biosanitaria de Granada, ibs.GRANADA, 18012 Granada, Spain; 3 Department of Anatomy and Embryology, Faculty of Medicine, University of Granada, 18071 Granada, Spain; 4 Aula del Mar CEI·Mar, Facultad de Ciencias, Universidad de Granada, 18071 Granada, Spain; pula@ugr.es

**Keywords:** cancer, nudibranchs, marine natural products

## Abstract

Nudibranchs have garnered increasing interest in biomedical research due to their complex chemical defense mechanisms, many of which are derived from their diet, including sponges, cnidarians, tunicates, and algae. Their remarkable ability to sequester dietary toxins and synthesize secondary metabolites positions them as a promising source of biologically active compounds with potential therapeutic applications, particularly in oncology. This study aimed to review and summarize the available literature on the bioactive potential of nudibranch-derived compounds, focusing mainly on their antitumor properties. Although research in this area is still limited, recent studies have identified alkaloids and terpenoids isolated from species such as *Dolabella auricularia*, *Jorunna funebris*, *Dendrodoris fumata*, and members of the genus *Phyllidia*. These compounds exhibit notable cytotoxic activity against human cancer cell lines, including those from colon (HCT-116, HT-29, SW-480), lung (A549), and breast (MCF7) cancer. These findings suggest that compounds derived from nudibranchs could serve as scaffolds for the development of more effective and selective anticancer therapies. In conclusion, nudibranchs represent a valuable yet underexplored resource for antitumor drug discovery, with significant potential to contribute to the development of novel cancer treatments.

## 1. Introduction

Cancer is one of the leading causes of morbidity and mortality worldwide. By 2022, the number of cancer diagnoses had reached 20 million, with projections estimating that it will rise to 35 million by 2050. In that same year, approximately 9.7 million cancer-related deaths were recorded, and 30% of premature deaths in individuals aged between 30 and 69 were related to cancer. Therefore, this situation is a challenge to increasing life expectancy in many countries [1]. This public health problem is mainly related to the delayed diagnosis of various types of cancer, often occurring after the metastasis development, as well as the limited effectiveness of conventional therapies due to tumor resistance and low drug specificity, which can lead to serious adverse effects [2,3,4,5]. These factors are thought to be the causes of more than 90% of cancer-related deaths [6,7]. Consequently, it is necessary to search for new drugs to develop novel therapies or enhance existing ones. So far, the search for new drugs has primarily focused on natural resources, including plants, terrestrial fungi, and bacteria. For instance, some molecules currently used in conventional therapies, such as paclitaxel, have been isolated from plants [8].

The search for new bioactive compounds has extended to the oceans, which cover approximately 70% of the Earth’s surface and are among the most biodiverse ecosystems. Many marine organisms have adapted to extreme environmental conditions, leading to the production of secondary metabolites with high bioactivity. Marine natural products (MNPs) are characterized by a great chemical diversity, which gives them significant pharmacological potential [9]. Although there are structural similarities between terrestrial and marine metabolites, notable differences have been observed. Marine metabolites have longer carbon chains, larger ring systems with more nitrogen atoms, fewer oxygen atoms, and a higher presence of halogen atoms compared with terrestrial metabolites [9].

### 1.1. Marine-Derived Drugs

To date, at least twenty marine-derived drugs have been approved by the Food and Drug Administration (FDA) for clinical use, many of which are indicated for antitumor treatment [10], while thirty-three more are currently undergoing clinical trials [9]. Most of these drugs have been derived from marine invertebrates such as mollusks, sea squirts, and sponges. Examples include cytarabine, a synthetic analog of compounds originally isolated from the marine sponge *Tectitethya crypta*; trabectedin, derived from the ascidian *Ecteinascidia turbinata*; eribulin mesylate, a synthetic analog of halichondrin B, which was isolated from the marine sponge *Halichondria okadai*; or brentuximab vedotin, an antibody–drug conjugate composed of a monoclonal antibody conjugated to the cytotoxic agent monomethyl auristatin E (MMAE), a synthetic analog of dolastatin 10 (*Dolabella auricularia*) (Table 1). However, the approved drugs have been associated with limitations that reduce their efficacy. For example, resistance to cytarabine has been observed in certain leukemia cell lines, and its efficacy is limited in treating other types of cancer [10]. Similarly, resistant cell lines have also been reported with trabectedin [11]. Both eribulin mesylate and brentuximab vedotin target actively dividing cells, but cancer stem cells, which are responsible for metastasis and tumor relapse, can evade this mechanism of action by entering a reversible dormant state [12]. In addition, these marine-derived drugs are associated with numerous side effects, indicating a lack of selectivity [10]. These limitations highlight the need for further research on new marine natural products.

### 1.2. Marine Invertebrates as a Source of MNPs

Marine invertebrates are increasingly recognized as a promising source of MNPs due to their significant genetic diversity, which implies high chemical diversity [13]. Among the most studied marine invertebrates are the phyla Porifera (sponges) and Cnidaria (e.g., corals, sea anemones, and jellyfish), due to their production of cytotoxic, antimicrobial, and antitumor compounds with promising pharmaceutical applications [14,15]. These bioactive compounds comprise a variety of unique chemical structures, including alkaloids [16] (e.g., nortopsentins [17,18]), terpenoids (e.g., sesquiterpenoids [19], diterpenoids [20]), peptides [21], and polyketides [22]. Most marine invertebrates are either sessile or slow-moving, soft-bodied organisms that lack physical defense mechanisms such as shells or spines or the ability to rapidly escape predators. Consequently, their primary strategy for defense involves the production of secondary metabolites and toxins [23]. These metabolites can have many functions, including deterring predators, inhibiting the growth of competing organisms, and adapting to harsh environmental conditions such as variable salinity, pressure, and temperature [24].

In this context, nudibranchs, which are a diverse group of gastropod mollusks, have developed a unique defense strategy: many species can sequester and store toxins or secondary metabolites from their prey, such as sponges, cnidarians (e.g., anemones and jellyfish), and other invertebrates, using these compounds for their chemical defense against predators [25]. The study of nudibranchs as a source of MNPs could help to overcome one of the main challenges of marine bioprospecting: the low availability and limited quantities of bioactive compounds in sessile marine organisms like sponges and anemones [26]. By feeding on these organisms and accumulating their chemical compounds, nudibranchs act as natural “bioaccumulators” of secondary metabolites. As a result, they can contain higher and more accessible concentrations of these bioactive compounds, making them easier to study and potentially use in pharmaceuticals. Moreover, the ability of some nudibranchs to synthesize their own chemical modifications of these metabolites opens up opportunities for discovering new molecules with unique properties [27].

Therefore, this review aims to provide a comprehensive and updated overview of the potential of nudibranchs as a source of MNPs with antitumor activity. It focuses on the main taxonomic groups of nudibranchs that have been studied so far, highlighting the methods used to isolate and characterize their bioactive metabolites. The review pays special attention to the cytotoxic or antiproliferative effects of these compounds on cancer cell lines, as well as the underlying mechanisms of action. Furthermore, it discusses current limitations in the field and outlines future research directions.

## 2. Nudibranchs

Nudibranchs (order Nudibranchia) are a diverse group of marine gastropod mollusks comprising about 4700 described species distributed worldwide. These organisms are characterized by their striking aposematic coloration and the lack of an external shell, which sets them apart from many other gastropods. Due to the absence of an external structure or other physical defenses, nudibranchs have developed unique chemical defense strategies. These strategies often involve the accumulation and modification of dietary secondary compounds and toxins, or the de novo synthesis of bioactive metabolites, which help to protect them from predators [25,28]. Nudibranchs primarily feed on other marine invertebrates, especially sessile organisms such as sponges, cnidarians, tunicates, and bryozoans [25]. Many of these prey organisms are known to be sources of MNPs, including sponges, tunicates, and anemones [29,30]. Nudibranchs possess the ability to capture metabolites synthesized by their prey, allowing them to accumulate and, in some cases, modifying these compounds for their own chemical defense purposes [25,27].

**Figure 1:**
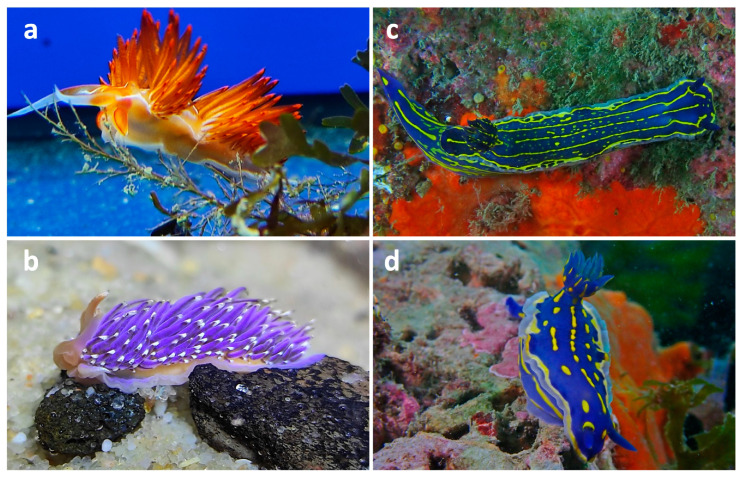
Nudibranch morphological groups: cladobranch nudibranchs including *Nemesignis banyulensis* (**a**) and *Facelina vicina* (**b**) and dorid nudibranchs including *Felimare picta* (**c**,**d**).

Nudibranchs can be classified into two main groups based on their morphology: dorids (suborder *Doridina*) and cladobranchs (suborder *Cladobranchia*) (Figure 1) [31]. Cladobranchs are characterized by the presence of numerous dorsal extensions called cerata, which serve various functions including respiration, digestion, and defense. These extensions act as secondary gills, increasing the surface area for gas exchange. In addition, many cerata contain specialized structures known as cnidosacs, which store functional nematocysts (stinging cells) obtained from their cnidarian prey, allowing nudibranchs to repurpose them for defensive use [32,33]. Notably, bioactive compounds and toxins are often concentrated in the distal regions of these cerata, providing an effective chemical defense against predators [34,35,36]. In contrast, dorids lack cerata and instead rely on their mantle for both protection and chemical defense. The mantle, which forms the dorsal surface of the body, is often thickened and glandular, containing numerous defensive glands (mantle dermal formations, or MDFs) where bioactive secondary metabolites may be stored or synthesized [37,38]. These compounds are distributed throughout the mantle tissue to enhance exposure to potential predators [39]. Many of these bioactive substances are classified as terpenoids, polyketides, and alkaloids, often derived from the nudibranchs’ sponge-based diet [22]. Dorids also possess a branchial plume consisting of a cluster of gills located on the posterior dorsal surface [25].

In addition, nudibranch species can consume either specialized or generalist diets [40]. For example, most dorid nudibranchs are known to primarily feed on sponges, whereas cladobranch nudibranchs predominantly consume cnidarians such as anemones [25].

The ability of nudibranchs to accumulate MNPs from their prey gives them a significant advantage in marine bioprospecting. One of the major challenges of working with sessile organisms such as sponges is that they often contain very low concentrations of bioactive compounds, which makes it difficult to isolate and evaluate them pharmacologically [26,41]. In contrast, nudibranchs can act as natural concentrators of these metabolites, allowing for much higher and more accessible levels to be found within their tissues. Furthermore, the compartmentalization of these MNPs into specific anatomical structures, like the distal tips of the cerata in cladobranch nudibranchs, makes it easier to extract and purify these compounds for research and potential therapeutic applications [25].

## 3. Nudibranchs as a Source of Antitumor Molecules

**Figure 2:**
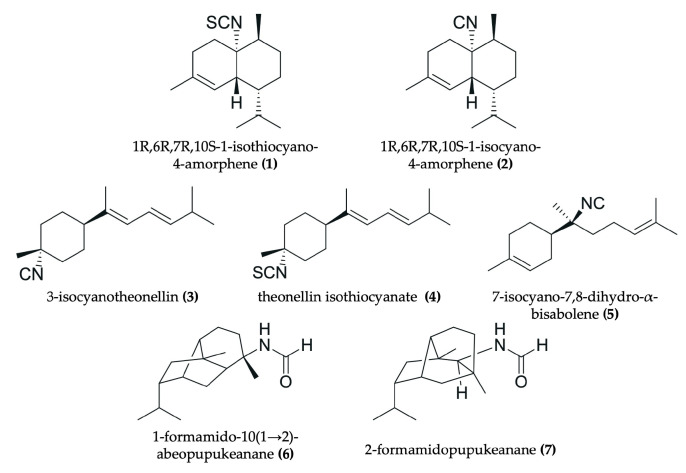
Chemical structures of antitumor compounds isolated from *Phyllidiella pustulosa* (**1−3**) and *Phyllidia coelestis* (**3−7**).

**Figure 3:**
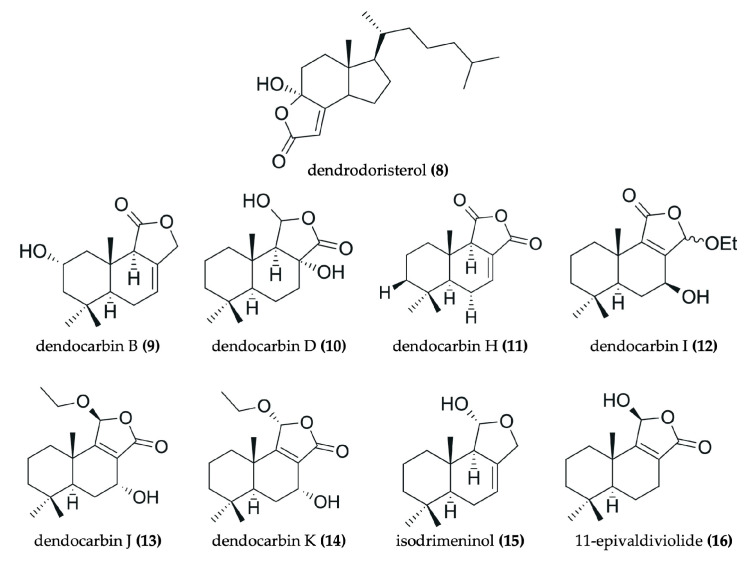
Chemical structures of antitumor compounds isolated from *Dendrodoris fumata* (**8**) and *D. carbunculosa* (**9−16**).

**Figure 4:**
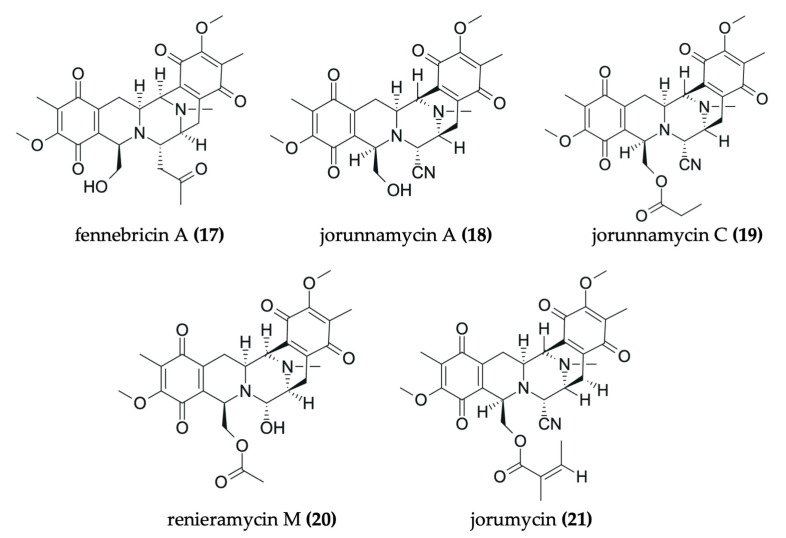
Chemical structures of antitumor compounds isolated from *Jorunna funebris* (**17−21**).

**Figure 5:**
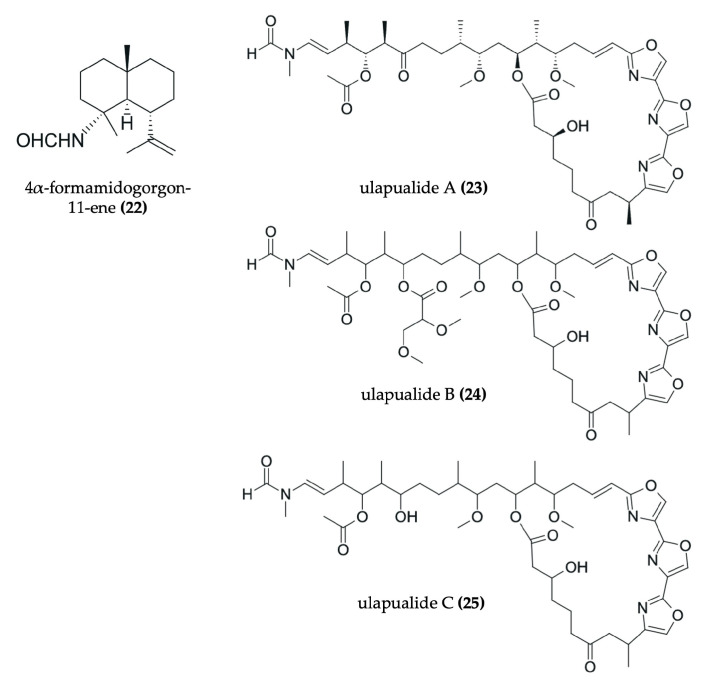
Chemical structures of antitumor compounds isolated from *Hexabranchus sanguineus* (**22−25**).

**Figure 6:**
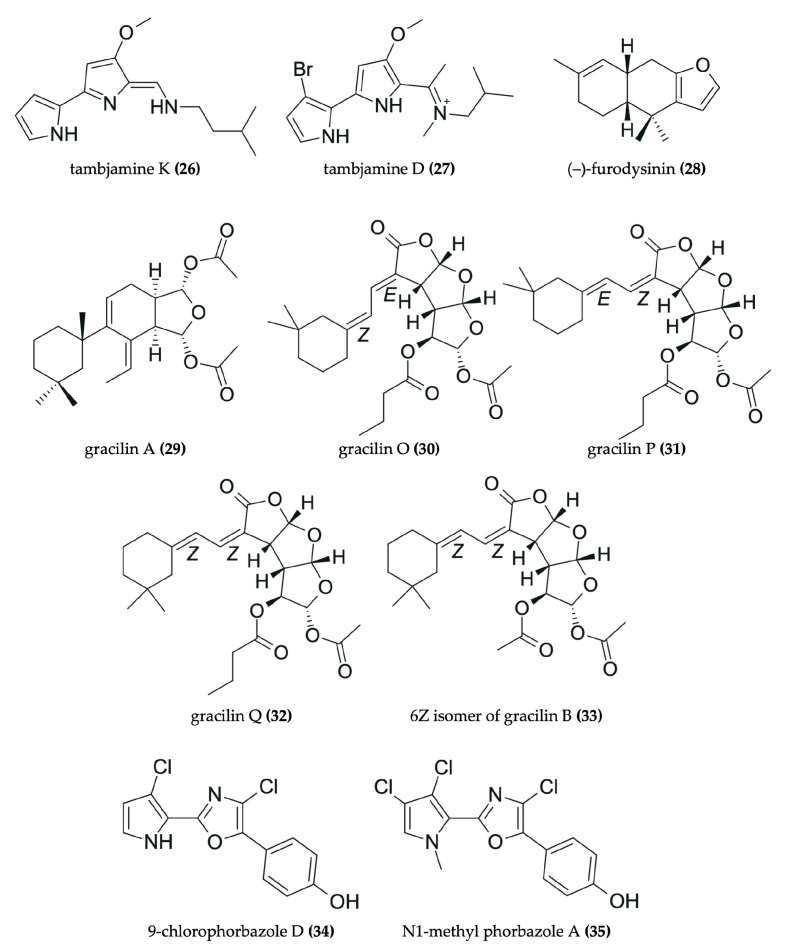
Chemical structures of antitumor compounds isolated from *Tambja ceutae* (**26**), *Tambja eliora* (**27**), *Hypselodoris infucata* (**28**), *Goniobranchus splendidus* (**29−33**), and *Aldisa andersoni* (**34**, **35**).

**Figure 7:**
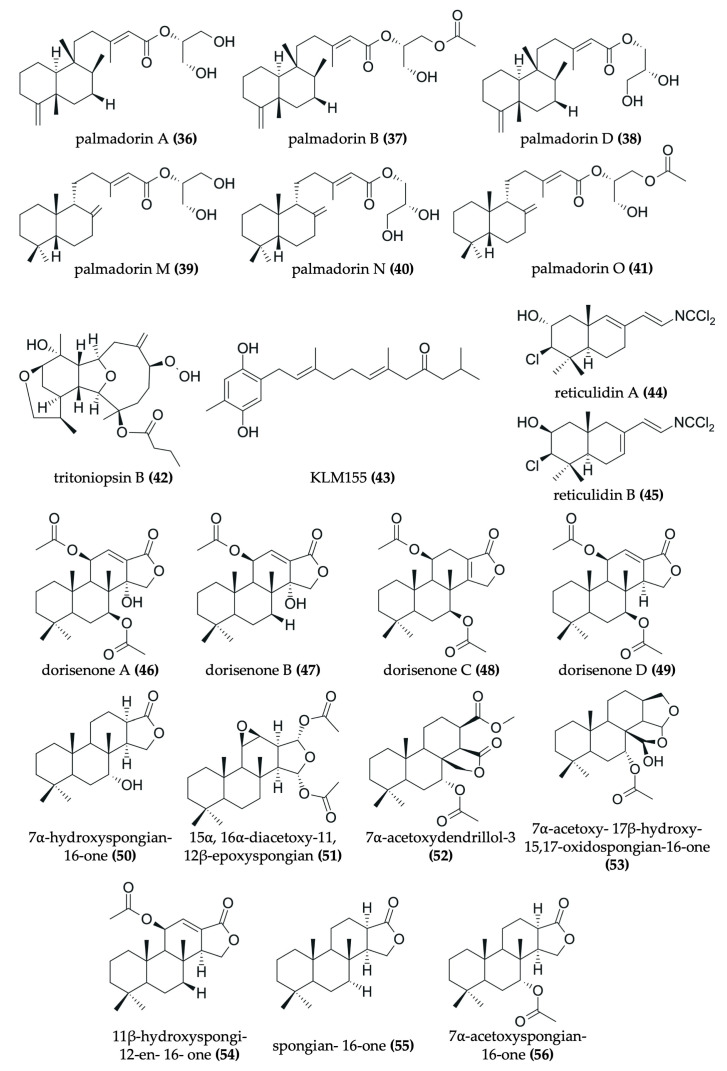
Chemical structures of antitumor compounds isolated from *Austrodoris kerguelenensis* (**36−41**), *Tritoniopsis elegans* (**42**), *Leminda millecra* (**43**), *Reticulidia fungia* (**44**, **45**), and *Chromodoris obsoleta* (**46−56**).

Nudibranchs are emerging as promising candidates in the search for novel, pharmacologically active agents for cancer therapy through the sequestration and modification of dietary bioactive compounds for use in chemical defense. Despite being poorly studied, numerous investigations have identified a wide range of secondary metabolites in nudibranchs that possess cytotoxic properties, many of which show promising potential as antitumor agents (Figure 2, Figure 3, Figure 4, Figure 5, Figure 6 and Figure 7, Table 2). To date, research has primarily focused on various species of nudibranchs within the dorid morphological group, likely because dorids are more prevalent than cladobranchs, comprising approximately 2000 of the 4700 known nudibranch species [40]. In particular, the most studied genera include *Phyllidia*, *Phyllidiella* [42,43,44,45], *Dendrodoris* [46,47], *Jorunna* [48,49,50], *Hexabranchus* [51,52,53], and *Tambja* [54,55,56].

Most published research has concentrated on analyzing the entire body of the nudibranch. However, some authors suggest that dissecting the mantle and viscera of nudibranchs for separate analysis would be beneficial, as dorid species are known to store defensive compounds in their external surface. Indeed, Nuzzo et al. observed higher concentrations of the cytotoxic phorbazole alkaloids (**34**, **35**) in the mantle compared with the viscera of the dorid nudibranch *Aldisa andersoni* [57]. In the case of *Phyllidia coelestis*, Jaisamut et al. focused on studying the mantle protrusions called tubercles, where they described several bridged tricyclic sesquiterpenes (**6**, **7**) [45].

Conversely, Wu et al. processed the mantle and viscera separately for the dorid species *Phyllidiella pustulosa*, but combined them for *Phyllidia coelestis*, because they found a similar composition using thin-layer chromatography (TLC) [44]. The cytotoxic sesquiterpene 3-isocyanotheonellin (**3**) was present in similar quantities in both tissues of *P. pustulosa*, and it was also isolated from *P. coelestis* extract. Two additional bisabolene-type sesquiterpenoids (**4**, **5**) with antitumor potential were described in *P. coelestis.* Interestingly, structural analogs of these compounds were also identified in their sponge prey *Axinissa variabilis* [44], suggesting a dietary origin of these metabolites. A similar situation was observed with the dorid nudibranch *Jorunna funnebris* and its sponge prey *Xestospongia* sp., as described by Huang et al. [48]. They identified numerous alkaloids in both the mantle and viscera of *J. funnebris*, including five metabolites that were also found in *Xestospongia* sp., reinforcing the idea of a predator–prey relationship between these two species. Among these compounds, the cytotoxic alkaloid fennebricin A (**17**) was exclusively found in the nudibranch tissues, although it is structurally related to two inactive compounds identified in the sponge, referred to as fennebricins C and D [48]. These findings suggest that prey-derived metabolites, potentially modified, can be present in both the viscera and more exposed body regions.

While the mantle generally contains a wider variety of metabolites than the viscera, some species have shown a greater number of metabolites in the internal organs [58]. Indeed, some researchers focus on these viscera to study the presence of active metabolites. For example, Shen et al. conducted a detailed chemical investigation on the internal organs of *Hexabranchus sanguineus* [51]. They described thirteen new sesquiterpenoids (sanyagunins A–H, sanyalides A–C, and sanyalactams A and B), along with eleven known compounds. Furthermore, several of these metabolites, including the cytotoxic 4α-formamidogorgon-11-ene (**22**), had previously been found in sponges of the genera *Dysidea* and *Halichondria*, suggesting they might be of dietary origin [51]. This concept of dietary origin for nudibranch metabolites is further supported by the work of Ciavatta et al. [59]. They isolated four diterpenes, tritoniopsins A–D, from both the nudibranch *Tritoniopsis elegans* and its prey, the soft coral *Cladiella krempfi*, confirming that these compounds were acquired through the nudibranch’s diet.

Interestingly, other research on nudibranchs has focused on studying egg masses from nudibranchs as sources of active metabolites, as in the case of *Hexabranchus sanguineus* and *Jorunna funebris* [49,52,53]. Nudibranch egg masses are often brightly colored and exposed in the marine environment, making them highly vulnerable to predation [60]. Consequently, it is hypothesized that nudibranchs incorporate defensive secondary metabolites into their egg masses to protect the developing embryos from predators and pathogens [61]. Indeed, the egg masses can be a concentrated source of these defensive compounds, making them easier to isolate and study compared with extracting them from the whole nudibranch or its prey.

### 3.1. Extraction and Isolation Methods for Obtaining Bioactive Extracts and Compounds from Nudibranchs

A literature review revealed that the most commonly used extraction methods for studying the antitumor potential of nudibranchs typically involve solvents such as acetone [41,42,44,48,50,54,57,59,62,63,64,65], methanol [46,49,53,55], or ethanol [47], often assisted by ultrasound, as well as mixtures of organic solvents such as methanol and dichloromethane (1:1, *v*:*v*) [43,51,52,66], methanol and chloroform (1:3, *v*:*v*) [67], or methanol and ethyl acetate (1:1, *v*:*v*) [45]. Methanol–dichloromethane extracts were used to obtain hydrophobic compounds [68], while acetone extracts were employed to isolate metabolites of intermediate polarity [69]. Most studies then subjected the obtained extracts to a liquid–liquid extraction (LLE) process employing a polar solvent (e.g., water or n-butanol) alongside an apolar solvent (e.g., dichloromethane, diethyl ether, n-hexane, or ethyl acetate) [70]. However, Huong et al. opted for LLE with two apolar solvents, ethyl acetate and n-hexane, likely to separate highly hydrophobic compounds into the n-hexane layer and compounds of intermediate polarity into the ethyl acetate phase [46]. In general, the organic extracts were purified through repeated column chromatography with silica gel, Sephadex LH-20, or C18 reversed-phase high-performance liquid chromatography (RP-HPLC) as stationary phases to obtain fractions and isolate the compounds of interest. Following chromatographic purification, the isolated compounds were characterized by nuclear magnetic resonance (NMR) spectroscopy to determine their chemical structures.

### 3.2. Chemical Structures of Antitumor Compounds Isolated from Nudibranchs

A high chemical diversity was observed among the antitumor compounds derived from nudibranchs. Terpenes, primarily sesquiterpenes and diterpenoids, and alkaloids represent the main types of bioactive molecule found in nudibranchs.

#### 3.2.1. Examples of Antitumor Sesquiterpenes in Nudibranchs

For instance, bisabolane-type sesquiterpenoids, such as 3-isocyanotheonellin (**3**) (from *Phyllidiella pustulosa* and *Phyllidia coelestis*), theonellin isothiocyanate (**4**) (from *P. coelestis*), and 7-isocyano-7,8-dihydro-α-bisabolene (**5**) (from *P. coelestis*) have been identified [44] (Figure 2). Structurally, these compounds are monocyclic sesquiterpenoids, specifically belonging to the bisabolane type. This means they are derived from a 15-carbon skeleton featuring a 6-membered ring to which a branched isoprenoid chain is attached. Furthermore, other sesquiterpenes have been isolated from *P. pustulosa*, including the isothiocyanate sesquiterpene 1R,6R,7R,10S-1-isothiocyanato-4-amorphene (**1**) and the isocyanate sesquiterpene 1R,6R,7R,10S-1-isocyanato-4-amorphene (**2**) [42] (Figure 2). Both compounds are bicyclic sesquiterpenes that belong to the amorphene class, a family of sesquiterpenes characterized by a decalin structure (two fused six-membered rings) with an isopropyl group. In addition, bridged sesquiterpenes 1-formamido-10(1 → 2)-abeopupukeanane (**6**) and 2-formamidopupukeanane (**7**) were also isolated from the nudibranch *P. coelestis* [45] (Figure 2). Specifically, these compounds possess a complex, rigid, and highly fused polycyclic system characteristic of pupukeanane-type structures along with a formamido (-NHCHO) functional group.

Drimane sesquiterpenes have also been described in nudibranchs. The drimane lactone sesquiterpenes dendocarbin B (**9**), D (**10**), H–K (**11**–**14**), and 11-epivaldiviolide (**16**), along with the drimane sesquiterpenoid isodrimeninol (**15**), were isolated from the nudibranch *Dendrodoris carbunculosa* [47] (Figure 3). These compounds share a fundamental drimane tricyclic carbon skeleton, typically featuring two six-membered rings and a fused five-membered lactone ring (or furan in the case of isodrimeninol), and they are all highly oxygenated. Moreover, the gorgonane-type sesquiterpenoid 4α-formamidogorgon-11-ene (**22**), characterized by a bicyclic structure with a NH-OH moiety, was isolated from the nudibranch *Hexabranchus sanguineus* [51] (Figure 5). Another example includes a tricyclic furanosesquiterpene, furodysinin (**28**), which was isolated from the nudibranch *Hypselodoris infucata* [62] (Figure 6). Finally, two bicyclic carbonimidic dichloride sesquiterpenes, featuring a rare functional group (N = CCl_2_), were isolated from the nudibranch *Reticulidia fungia*: reticulidin A (**44**) and reticulidin B (**45**) [65] (Figure 7).

#### 3.2.2. Examples of Antitumor Diterpenes in Nudibranchs

Spongian diterpenoids are particularly representative examples. These include dorisenone A–D (**46**–**49**); 7α-hydroxyspongian-16-one (**50**); 15α,16α-diacetoxy-11,12β-epoxyspongian (**51**); 7α-acetoxy-17β-hydroxy-15,17-oxidospongian-16-one (**53**); 7α-acetoxy-17β-hydroxy-15,17-oxidospongian-16-one (**53**); 11β-hydroxyspongi-12-en-16-one (**54**); spongian-16-one (**55**); and 7α-acetoxyspongian-16-one (**56**), all of which were isolated from *Chromodoris obsoleta* [67] (Figure 7). Characteristically, these compounds possess tetracyclic structures. Specifically, one of their rings resembles a furan with a carbonyl group, and at least one ring is not oxygenated. Furthermore, an acetyl group is consistently present, with the exceptions of 7α-hydroxyspongian-16-one (**50**) and spongian-16-one (**55**). Additionally, the palmadorin A (**36**), B (**37**), D (**38**), M (**39**), N (**40**), and O (**41**) diterpenoids, isolated from *Austrodoris kerguelensis* [66], are noteworthy (Figure 7). These compounds are glyceride esters, meaning an ester is attached to a bicyclic moiety. Another interesting diterpenoid is tritoniopsin B (**42**), isolated from *Tritoniopsis elegans* [59]. This compound is a cladiellane-based diterpenoid, where a nine-membered ring is attached to a diterpene moiety. Moreover, gracilins A (**29**), O (**30**), P (**31**), and Q (**32**), along with the 6Z isomer of gracilin B (**33**), serve as further examples of norditerpenes isolated from the nudibranch *Goniobranchus splendidus* [63] (Figure 6). These are complex diterpenoids characterized by an oxygenated tricyclic main structure to which an additional five-membered ring is attached. In contrast to gracilin A (**29**), gracilins O (**30**), P (**31**), and Q (**32**) and the 6Z isomer of gracilin B (**33**) are more highly oxygenated.

#### 3.2.3. Examples of Antitumor Alkaloids in Nudibranchs

Alkaloids are also a representative class of compounds found in nudibranchs. Among them, isoquinolinequinone alkaloids are the most common type. These include fennebricin A (**17**), jorunnamycin A (**18**) and C (**19**), renieramycin M (**20**), and jorumycin (**21**), all of which were isolated from *Jorunna funebris* [48,49,50] (Figure 4). Structurally, these molecules consist of five six-membered rings, with a nitrogen atom bearing a methyl group projecting backwards. Furthermore, other types of alkaloids have also been identified. For instance, tambjamine K (**26**) and tambjamine D (**27**) were isolated from nudibranchs belonging to the genus *Tambja* [54,55,56] (Figure 6). These are classified as methoxypyrrolic alkaloids due to the presence of a methoxy group in one of their rings. Additionally, indole-containing alkaloids known as phorbazoles, such as 9-chlorophorbazole D (**34**) and N1-methylphorbazole A (**35**), have been discovered in the nudibranch *Aldisa andersoni* [57] (Figure 6).

#### 3.2.4. Other Types of Antitumor Compounds in Nudibranchs

Beyond terpenes and alkaloids, other types of natural products have also been isolated from nudibranchs, further highlighting their chemical diversity. For example, a tricyclic steroid with a long aliphatic chain, dendrodoristerol (**8**), was isolated from the nudibranch *Dendrodoris fumata* [46] (Figure 3). Similarly, a toluhydroquinone, KLM155 (**43**) isolated from the nudibranch *Leminda millecra* [64] (Figure 7), features one phenolic ring and one isoprenoid chain, resembling the meroterpenoids found in other marine organisms [71]. Furthermore, compounds not easily classified within the aforementioned groups have also been identified in nudibranchs. These include the tris-oxazole macrolides, ulapualides A–C (**23**–**25**) (Figure 5), which were isolated from the egg masses of *Hexabranchus sanguineus* [52,53]. Notably, these compounds are characterized by a large aliphatic moiety and a large ring containing three oxygenated pyrrole rings.

In addition to classifying compounds, examining the structural features of these marine natural products (MNPs) isolated from nudibranchs reveals several recurring patterns. Shang et al. reported that other MNPs often feature halogen atoms, long chains, and large rings (exceeding six members), as well as a higher count of nitrogen atoms and a lower count of oxygen atoms [70]. To illustrate, halogen atoms are indeed present in two phorbazole alkaloids: 9-chlorophorbazole (**34**) and N1-methylphorbazole (**35**). In these compounds, the chlorine atoms are specifically bound to the two five-membered rings of these alkaloids. Similarly, long chains are a characteristic of the ulapualides A–C (**23**–**25**), the toluhydroquinone KLM155 (**43**), and the bisabolane sesquiterpenoids (**3**–**5**). Furthermore, large rings are also observed, for example, in the cladiellane-based diterpenoid tritoniopsin B (**42**), which features a nine-membered ring. Regarding nitrogen content, some compounds do contain more than one nitrogen atom, including the previously reported alkaloids (**17**–**21**, **26**, **27**, **34**, **35**). However, it should be noted that many compounds also possess more than two oxygen atoms, such as the gracilins (**29**–**33**) and dorisenones (**46**–**49**) diterpenoids. Even more, the isoquinolinequinone alkaloids (**18**–**21**) are highly oxygenated. Therefore, the generalization of a smaller number of oxygen atoms cannot be broadly applied to all MNPs from nudibranchs. Finally, the presence of oxygenated substituents in some MNPs might be crucial to their biological activity. For instance, the hydroxyl group directly attached to one of the rings of fennebricin A (**17**) appears to be crucial to its bioactivity [48].

### 3.3. Antitumor Potential of Nudibranch-Derived Extracts and Compounds Against Cancer Cell Lines

Numerous extracts and compounds derived from nudibranchs have been reported to exhibit in vitro antitumor activity. A wide range of tumor cell lines has been used to evaluate the cytotoxicity of these derivatives. These include human colorectal (HCT-116, HT-29, HGUE-C1, SW-480, Caco-2, HCT-8), pancreatic (Panc-1, Capan-1), lung (A549, LU-1, QG56, H1975, H1299), breast (MCF-7, MDA-MB-231), prostate (LNCap, DU145), liver (HepG2, SNU-398), kidney (768-0), cervical (HeLa, KB, ME180, SiHa), gastric (AGS), esophageal (WHCO1, WHCO6), leukemia (HL-60, L1210, CEM, HEL), melanoma (SKMEL-28, MEL28), oligodendrocytoma (Hs683), and glioblastoma (U373) tumor cell lines as well as murine bladder cancer (NBT-T2), lymphocytic leukemia (P388), glioma (C6), and melanoma (B16) cell lines. To assess the selectivity and toxicity of the compounds toward healthy cells, non-tumorigenic cell lines such as CCD-18Co (normal human colon fibroblasts), MCF-12A (nonmalignant human breast epithelial cells), H9c2 (embryonic rat heart tissue), 3T3-L1 (embryonic mouse fibroblasts), and V79 (Chinese hamster lung fibroblast) have also been used.

Some studies have explored the antitumor properties of extracts from various nudibranchs such as *Dolabella auricularia*, *Phyllidia varicosa*, *Armina tigrina*, *A. maculata*, and *A. tricolorata*. However, the specific bioactive compounds responsible for this activity have not yet been isolated. Conversely, in most other studies, various compounds have been successfully isolated from nudibranch-derived extracts, with some demonstrating activity against different types of cancer cell lines.

#### 3.3.1. Nudibranchs-Derived Extracts with Antitumor Activity

A study published in 2019 by Ruiz-Torres et al. highlighted the potent cytotoxic effects of methanol–dichloromethane extracts obtained from *P. varicosa* and *D. auricularia* against several colorectal cancer (CRC) cell lines (HT-29, HGUE-C1, and SW-480) [43]. Notably, the extract from *D. auricularia* exhibited a stronger cytotoxic effect, with IC_50_ values ranging from 0.1 to 0.2 µg/mL, compared with *P. varicosa*, which showed IC_50_ values of 9.3, 78.8, and 13 µg/mL, respectively. The extract from *P. varicosa* was predominantly composed of long-chain fatty acids and lyso-PAF. Additionally, several bioactive compounds with previously reported antiproliferative properties were identified, including the diterpene spongian-16-one, the chlorophyll derivative ethyl pheophorbide A, the macrocyclic polyketide palmadorin A, and the indole-based diterpene rhizovarin D. Similarly, the *D. auricularia* extract contained high levels of spongian-16-one and the porphyrin derivative pyropheophorbide A, both of which have been associated with antiproliferative properties [43]. In a follow-up study published in 2021, the same authors confirmed the antitumor potential of the methanol–dichloromethane extract from *D. auricularia*, demonstrating certain selectivity toward tumor cells. Specifically, the extract exhibited an IC_50_ value of 1.01 µg/mL against the HCT-116 CRC cell line compared with an IC_50_ value of 15.04 µg/mL for the non-tumor colon fibroblast line CCD-18Co [72]. In contrast, Gomes et al. reported a lower cytotoxic effect for acetone extracts obtained from *A. tigrina*, *A. maculata*, and *A. tricolorata* against stomach (AGS) and lung (A549) cancer cell lines [41]. The extract from *Armina tigrina* exhibited IC_50_ values of approximately 69 µg/mL in both cell lines, whereas *A. maculata* showed IC_50_ values ranging from 200 to 500 µg/mL, and *A. tricolorata* exceeded 500 µg/mL. The observed antitumor activity has been associated with the presence of polyunsaturated fatty acids (PUFAs), particularly n-3 fatty acids such as docosahexaenoic acid (C22:6n-3) and eicosapentaenoic acid (C20:5n-3), as well as arachidonic acid (C20:4n-6) [73]. The antiproliferative potential of PUFAs has been previously described in A549 lung cancer cells [74,75].

#### 3.3.2. Antitumor Compounds Isolated from *Phyllidiella pustulosa* and *Phyllidia coelestis*

Various acetone extracts derived from *Phyllidiella pustulosa* have been purified to isolate the cytotoxic sesquiterpenes (1R,6R,7R,10S-1-isothiocyano-4-amorphene) (**1**), (1R,6R,7R,10S-1-isocyano-4-amorphene) (**2**) [42], and 3-isocyanotheonellin (**3**) [44] (Figure 2). Similar compounds were obtained from *Phyllidia coelestis* after acetone extraction and purification: isocyanotheonellin (**3**); theonellin isothiocyanate (**4**), and 7-isocyano-7,8-dihydro-α-bisabolene (**5**) [44] (Figure 2). Compounds (**1**) and (**2**) obtained IC_50_ values of 15.6–18.8 µg/mL and 19.7–23.7 µg/mL, respectively, in HCT-116 (CRC), NBT-T2 (murine bladder cancer), and Panc-1 (pancreatic cancer) cell lines. Likewise, 3-isocyanotheonellin (**3**) exhibited significant cytotoxicity against various cancer cell lines, including lung cancer A549 (IC_50_: 8.60 µM), CRC HT-29 (IC_50_: 3.35 µM), liver cancer SNU-398 (IC_50_: 0.5 µM), and pancreatic cancer Capan-1 (IC_50_: 1.98 µM) [44]. However, compounds (**4**) and (**5**) from *P. coelestis* seemed to be cytotoxic only in the SNU-398 cell line (IC_50_: 2.15 and 0.5 µM, respectively) [44]. Jaisamut et al. also described the extraction, isolation, and chemical characterization of bridged tricyclic sesquiterpenes 1-formamido-10(1 → 2)-abeopupukeanane (**6**) and 2-formamidopupukeanane (**7**) from tubercles of *P. coelestis* (Figure 2). Both compounds showed a potent antitumor activity against several cancer cell lines (HeLa, MCF-7, KB, and HT-29) with IC_50_ values of 0.13, 0.65, 2.4, and 6.8 µM for compound (**6**) and 0.07, 8.2, 1.2, and >20 µM for compound (**7**) [45].

#### 3.3.3. Antitumor Compounds Isolated from the *Dendrodoris* Genus

Regarding nudibranchs of the genus *Dendrodoris*, the antitumor potential of compounds isolated from two species, *D. fumata* and *D. carbunculosa*, has been studied. Firstly, the steroid dendrodoristerol (**8**) (Figure 3) was purified from the methanol extract of *D. fumata*. It showed moderate cytotoxicity with IC_50_ values ranging from 21 to 41 µM across a panel of tumor cell lines (HL-60, KB, LU-1, MCF-7, LNCaP, and HepG2) [46]. Secondly, *D. carbunculosa* was extracted with ethanol, partitioned with ethyl acetate, and then subjected to fractionation to obtain several compounds. Among these, we highlight those with antitumor potential against the P388 murine lymphoid neoplasm cell line. These include the sesquiterpene lactones dendocarbin B (**9**), D (**10**), H–K (**11**–**14**), and the sesquiterpenoid isodrimeninol (**15**) (Figure 3), with IC_50_ values of 10–17 μg/mL. Additionally, the drimane lactone 11-epivaldiviolide (**16**) showed an IC_50_ value of 3.2 μg/mL [47].

#### 3.3.4. Antitumor Compounds Isolated from *Jorunna funebris*

Another extensively studied species is *Jorunna funebris*. Three different investigations have described various isoquinoline alkaloids from samples of its mantle, viscera, egg ribbons, and/or mucus, extracted with either acetone or methanol. Among these, several compounds have demonstrated potent in vitro antitumor activity: fennebricin A (**17**), with IC_50_ values of 6.2 µM in A549 and 2.5 µM in HL-60 [48]; jorunnamycin A (**18**) and C (**19**), which exhibited remarkable IC_50_ values (13 and 1.5 nM in HCT-116, 59 and 2.8 nM in QG56, and 29 and 0.32 nM in DU145 cell lines, respectively); renieramycin M (**20**), showing IC_50_ values of 7.9 nM in HCT-116 and 19 nM in QG56 [49]; and jorumycin (**21**), with a IC_50_ value of 12.5 ng/mL in P388, A549, HT-29, and MEL28 [50] (Figure 4).

#### 3.3.5. Antitumor Compounds Isolated from *Hexabranchus sanguineus*

Nudibranchs of the species *Hexabranchus sanguineus* have garnered significant interest, not only for the active compounds found in adult organisms but also for those described in their egg masses. A recent investigation by Shen et al. reported the presence of the cytotoxic gorgonane-type sesquiterpenoid: 4α-formamidogorgon-11-ene (**22**) (Figure 5). This compound showed activity in several tumor cell lines, including H1975 (IC_50_: 0.87 μM), MDA-MB-231 (IC_50_: 1.04 μM), A549 (IC_50_: 1.95 μM), and H1299 (IC_50_: 1.34 μM) [51]. Furthermore, Parrish et al. complemented a previous study by Roesener & Scheuer, which described the presence of several bioactive compounds (ulapualides A–C) in the egg masses of this nudibranch. In the initial study, ulapualide A (**23**) and B (**24**) demonstrated antitumor activity against the murine lymphocytic leukemia cell line L1210 (IC_50_: 0.01–0.03 μg/mL) [53]. Subsequently, ulapualide A–C (**23**–**25**) (Figure 5) isolated from these *H. sanguineus* egg masses exhibited high cytotoxicity in the tumor cell lines 768-0, DU-145, MDA-MB-231, and A549 (IC_50_ values: 0.24–1.3 μM) [52].

#### 3.3.6. Antitumor Compounds Isolated from the *Tambja* Genus

Tambjamines A, C, and D have been identified as the predominant metabolites within the tissues of the nudibranchs *Tambja stegosauriformis* and *Tambja brasiliensis* and their prey, the bryozoans *Virididentula dentata.* These compounds are likely the primary chemical defenses for these marine invertebrates [76]. The pyrrole alkaloids tambjamine K (**26**) and D (**27**) (Figure 6), isolated from *Tambja ceutae* and *Tambja eliora*, respectively, exhibited potent antitumor activity against several cancer cell lines [54,55,56]. In detail, tambjamine K (**26**) showed IC_50_ values of 14.6 and 14 µM against HeLa (cervical cancer) and C6 (rat glioma) cell lines, respectively. Meanwhile, tambjamine D (**27**) exerted comparable cytotoxicity against CEM (acute lymphoblastic leukemia), HL60 (myeloid leukemia), MCF7 (breast cancer), HCT-8 (colon cancer), and B16 (mouse melanoma) cell lines, with IC_50_ values of 12.2, 13.2, 13.2, 10.1, and 6.7 µg/mL, respectively. However, a notable drawback was their observed toxicity against non-tumor cell lines, including H9c2 (embryonic rat heart tissue), 3T3-L1 (embryonic mouse fibroblasts), and V79 (Chinese hamster lung fibroblast).

#### 3.3.7. Antitumor Compounds Isolated from Other Nudibranch Species

As noted, diterpenes and sesquiterpenes are common active compounds found in nudibranchs. Several diterpenoids with antitumor activity have been identified across various nudibranch species. For instance, gracilins A (**29**), O (**30**), P (**31**), and Q (**32**), along with the 6Z isomer of gracilin B (**33**) (Figure 6), isolated from *Goniobranchus splendidus*, showed potent activity with IC_50_ values less than 0.32 µg/mL in the HeLa S3 cell line [63]. *Austrodoris kerguelenensis* yields diterpenoid glyceride esters such as palmadorin A (**36**), B (**37**), D (**38**), M (**39**), N (**40**), and O (**41**) (Figure 7), which demonstrated IC_50_ values ranging from 4.9 to 16.5 μM in the HEL cell line [66]. Additionally, tritoniopsin B (**42**) (Figure 7), a cladiellane-based diterpene from *Tritoniopsis elegans*, exhibited toxicity in the Caco-2 colorectal cancer cell line as well as in non-tumor cell lines H9c2 and 3T3-L1 (IC_50_: 40–65 μM) [59]. Finally, various spongian diterpenoids (**46**–**56**) (Figure 7) from *Chromodoris obsoleta* also displayed significant activity, with IC_50_ values below 20 μg/mL in both L1210 and KB cell lines [67]. Regarding sesquiterpene-type compounds, (–)-furodysinin (**28**) (Figure 6) was isolated from *Hypselodoris infucata*, and reticulidins A (**44**) and B (**45**) (Figure 7) were isolated from *Reticulidia fungia*. While (–)-furodysinin (**28**) exhibited a relatively high IC_50_ against the HeLa cervical cancer cell line (102.7 µg/mL) [62], reticulidins A (**44**) and B (**45**) demonstrated potent cytotoxic effects on the KB cervical cancer cell line (IC_50_: 0.41 and 0.42 μg/mL, respectively) and the L1210 leukemia cell line (0.59 and 0.11 μg/mL, respectively) [65].

Despite showing higher IC_50_, the phorbazole alkaloids 9-chlorophorbazole D (**34**) and N1-methyl phorbazole A (**35**) (Figure 6) isolated from *Aldisa andersoni* also demonstrated cytotoxic activity against several cancer cell lines, including lung cancer (A549), breast cancer (MCF-7), melanoma (SKMEL-28), oligodendroglioma (HS683), and glioblastoma (U373), with IC_50_ values ranging within 18−29 μM and 19−34 μM, respectively. Notably, the A549, SKMEL-28, and U373 cell lines have exhibited resistance to proapoptotic stimuli [57]. In addition, KLM155 (**43**) (Figure 7) isolated from *Leminda millecra* demonstrated a potent antiproliferative effect, particularly against two esophageal cancer cell lines, WHCO1 and WHCO6, with IC_50_ values of 9.5 µM and 5.8 µM, respectively. However, it exhibited higher IC_50_ values against cervical cancer cell lines ME180 and SiHa (33.9 µM and >150 µM, respectively) as well as against the nonmalignant breast epithelial cell line MCF12 (32 µM) [64]. In comparison, positive controls using conventional chemotherapeutic agents showed lower IC_50_ values than most compounds isolated from nudibranchs, except for KLM155 (**43**) and the two phorbazole alkaloids (**34**, **35**), which showed comparable cytotoxic potential [57,64].

**Table 2:** Antitumor potential of extracts or isolated compounds from nudibranch species.

Material	Extraction and Isolation Methods	Isolated Compounds	Antitumor Potential (IC_50_ Value)	Mechanisms of Action and Other Bioactive Effects
*Phyllidiella pustulosa* [42]	(1) Extraction with acetone (2) LLE with dichloromethane and water (3) Organic phase was purified by silica gel chromatography and HPLC	Isothiocyanate sesquiterpene (1R,6R,7R,10S-1-isothiocyano-4-amorphene) (**1**); isocyano sesquiterpene (1R,6R,7R,10S-1-isocyano-4-amorphene) (**2**)	Panc-1: 18.8 (**1**) and 23.7 µg/mL (**2**) NBT-T2: 17.5 (**1**) and 20.5 µg/mL (**2**) HCT-116: 15.6 (**1**) and 19.7 µg/mL (**2**)	-
Mantle and viscera of *Phyllidiella pustulosa* or *Phyllidia coelestis* [44]	(1) Extraction in acetone using ultrasound (2) LLE with water, diethyl ether, and N-butanol (3) Ether extracts purified on repeated column chromatography (silica gel, Sephadex LH-20, RP-C18, and RP-HPLC)	Bisabolane-type sesquiterpenoids: 3-isocyanotheonellin (**3**) (*P. pustulosa* and *P. coelestis*); theonellin isothiocyanate (**4**) (*P. coelestis*), and 7-isocyano-7,8-dihydro-α-bisabolene (**5**) (*P. coelestis*)	A549: 8.6 (**3**) and >50 µM (**4**, **5**) HT-29: 3.35 (**3**) and >50 µM (**4**, **5**) Capan-1: 1.98 (**3**) and >50 µM (**4**, **5**) SNU-398: 0.5 (**3**), 2.15 (**4**), and 0.5 µM (**5**)	-
Tubercle of *Phyllidia coelestis* [45]	(1) Maceration in methanol and ethyl acetate (1:1, *v*:*v*) (2) Consecutive LLE with ethyl acetate, n-hexane, chloroform, n-butanol (3) Hexane extract subjected to repeated column chromatography (silica gel, Sephadex LH20, silica HPLC)	Bridged tricyclic sesquiterpenes: 1-formamido-10(1 → 2)-abeopupukeanane (**6**) and 2-formamidopupukeanane (**7**)	HeLa: 0.13 (**6**) and 0.07 µM (**7**) MCF-7: 0.65 (**6**) and 8.2 µM (**7**) KB: 2.4 (**6**) and 1.2 µM (**7**) HT-29: 6.8 (**6**) and > 20 µM (**7**)	-
*Phyllidia varicosa*; *Dolabella auricularia* [43]	Extraction with methanol–dichloromethane (1:1, *v*:*v*) at 4 °C for 24 h	-	HT-29: 9.3 (*P. varicosa*) and 0.1 µg/mL (*D. auricularia*) HGUE-C1: 78.8 (*P. varicosa*) and 0.1 µg/mL (*D. auricularia*) SW-480: 13 (*P. varicosa*) and 0.2 µg/mL (*D. auricularia*)	Colony formation; cell cycle arrest and apoptosis; ROS generation and mitochondrial membrane depolarization; DNA damage
*Dolabella auricularia* [72]	Extraction with methanol–dichloromethane (1:1, *v*:*v*) at 4 °C for 24 h	-	HCT-116: 1.01 µg/mL CCD-18Co: 15.04 µg/mL	ROS generation and activation ER stress; DNA damage; G_2_/M cell cycle arrest and apoptosis; ↓ colony formation, cell migration and invasion
*Dendrodoris fumata* [46]	(1) Extraction with methanol and ultrasound (2) LLE with water, n-hexane, and ethyl acetate (3) Both organic phases combined and separated on silica gel chromatography	Steroid: dendrodoristerol (**8**)	HL-60: 21.63 µM KB: 22.22 µM LU-1: 24.53 µM MCF-7: 41.19 µM LNCaP: 25.34 µM HepG2: 21.59 µM	Apoptosis
*Dendrodoris carbunculosa* [47]	(1) Extraction in ethanol (2) LLE with ethyl acetate and water; aqueous phase was further extracted with 1-butanol (3) Ethyl acetate extract subjected to repeated column chromatography (silica gel, HPLC)	Drimane sesquiterpenes: dendocarbin B (**9**), D (**10**), H–K (**11**–**14**); sesquiterpenoid isodrimeninol (**15**); and drimane lactone 11-epivaldiviolide (**16**)	P388: 3.2 (**16**) and 10–17 μg/mL (**9**–**15**)	-
Mantle or viscera *Jorunna funebris* [48]	(1) Extraction in acetone (2) LLE with diethyl ether and n-butanol (3) Ether extract subjected to repeated column chromatography (silica gel, Sephadex LH-20)	Isoquinolinequinone alkaloid: fennebricin A (**17**)	A549: 6.2 µM HL-60: 2.5 µM	NF-κB signaling pathway inhibition
Mantle, viscera, and egg ribbons of *Jorunna funebris* [49]	(1) Homogenized with phosphate buffer (pH 7) and treatment with 10% KCN (2) Maceration with methanol (room temperature, 24 h) (3) LLE with ethyl acetate and water (4) Ethyl acetate extracts subjected to repeated column chromatography (silica gel, Sephadex LH-20, C18 RP HPLC)	Isoquinoline alkaloids: jorunnamycin A (**18**) and C (**19**), renieramycin M (**20**)	HCT-116: 13.0 (**18**), 1.5 (**19**), and 7.9 nM (**20**) QG56: 59.0 (**18**), 2.8 (**19**), and 19.0 nM (**20**) DU145: 29.0 (**18**) and 0.32 nM (**19**)	-
Mantle and mucus of *Jorunna funebris* [50]	(1) Extraction in acetone (2) LLE with ethyl acetate and water (3) Ether extracts subjected to repeated column chromatography (Sephadex LH-20, silica gel TLC, HPLC)	Isoquinoline alkaloid: jorumycin (**21**)	P388: 12.5 ng/mL A549: 12.5 ng/mL HT-29: 12.5 ng/mL MEL28: 12.5 ng/mL	-
Internal organs of *Hexabranchus sanguineus* [51]	(1) Extraction with dichloromethane and methanol (1:1, *v*:*v*) (2) LLE with water and diethyl ether (3) Ether extract subjected to repeated column chromatography (silica gel, semipreparative HPLC)	Gorgonane-type sesquiterpenoid: 4α-formamidogorgon-11-ene (**22**)	H1975: 0.87 μM MDA-MB-231: 1.04 μM A549: 1.95 μM H1299: 1.34 μM	-
Egg mass of *Hexabranchus sanguineus* [52]	(1) Extraction with dichloromethane and methanol (1:1, *v*:*v*) (2) Extract subjected to C8 silica gel solid-phase extraction in increasing methanol and water content (3) A 75% methanol fraction subjected to repeated column chromatography (RP-HPLC, C18 column)	Ulapualide A–C (**23**–**25**)	768-0: 0.27 (**23**), 0.46 (**24**), and 1.3 μM (**25**) DU-145: 0.26 (**23**), 0.31 (**24**), and 0.8 μM (**25**) MDA-MB-231: 0.24 (**23**), 0.29 (**24**), and 0.67 μM (**25**) A549: 0.29 (**23**), 0.3 (**24**), and 0.64 μM (**25**)	-
Egg mass of *Hexabranchus sanguineus* [53]	(1) Extraction in methanol (2) LLE with hexane and then carbon tetrachloride (3) Carbon tetrachloride residue subjected to repeated column chromatography (C18 RP HPLC, silica HPLC)	Ulapualide A (**23**) and B (**24**)	L1210: 0.01–0.03 μg/mL	-
*Tambja ceutae* [54]	(1) Extraction with acetone (2) Ethereal soluble portions fractionated by LH-20 Sephadex column and SiO_2_ preparative TLC chromatography	Bromopyrrole alkaloid: tambjamine K (**26**)	Caco-2: 0.0035 µM HeLa: 14.6 µM C6: 14 µM H9c2: 2.7 µM 3T3-L1: 19 µM	-
*Tambja eliora* [55,56]	(1) Extraction in acetone and methanol using ultrasound (2) Extracts separated by chromatography on a Waters Sep-Pak silica gel column	4-methoxypyrrolic alkaloid: tambjamine D (**27**)	V79: 1.2 µg/mL CEM: 12.2 µg/mL HL60: 13.2 µg/mL MCF7: 13.2 µg/mL HCT-8: 10.1 µg/mL B16: 6.7 µg/mL	V79: Apoptosis; ROS generation; ↑ nitrite/nitrate and TBARS production; genotoxicity
*Armina tigrina; A. maculata;* and *A. tricolorata* [73]	Extraction with acetone	-	AGS: 68.75 (*A. tigrina*); 220.66 (*A. maculata*); >500 µg/mL (*A. tricolorata*) A549: 69.77 (*A. tigrina*); 250–500 (*A. maculata*); >500 µg/mL (*A. tricolorata*)	Anti-inflammatory effect
*Hypselodoris infucata* [62]	(1) Extraction in acetone using sonication (2) LLE with water and dichloromethane (3) Organic extract purified by normal phase chromatography eluting with 100% Hexane	Sesquiterpene: (–)-furodysinin (**28**)	HeLa: 102.7 µg/mL	-
*Goniobranchus splendidus* [63]	(1) Extraction in acetone (2) LLE with diethyl ether and water (3) Organic extract subjected to repeated column chromatography (silica gel, normal phase HPLC, or RP-HPLC)	Diterpenoids: gracilins A (**29**), O (**30**), P (**31**), and Q (**32**); 6Z isomer of gracilin B (**33**)	HeLa S3: <0.30 (**29**–**32**) and 0.32 µg/mL (**33**)	-
Mantle or viscera of *Aldisa andersoni* [57]	(1) Extraction in acetone and sonication (2) LLE with water and diethyl ether (3) Ether extract of the external part purified by C18 RP-HPLC	Alkaloids: 9-chlorophorbazole D (**34**) and N1-methyl phorbazole A (**35**)	A549: 29 (**34**) and 34 µM (**35**) MCF-7: 18 (**34**) and 25 µM (**35**) SKMEL-28: 22 (**34**) and 29 µM (**35**) Hs683: 25 (**34**) and 25 µM (**35**) U373: 19 (**34**) and 19 µM (**35**)	-
*Austrodoris kerguelenensis* [66]	(1) Extraction with dichloromethane and methanol (1:1, *v*:*v*) for 24 h (2) LLE with ethyl acetate and water (3) Lipophilic crude extract subjected to repeated column chromatography (silica gel, C18 HPLC)	Diterpenoid glyceride esters: palmadorin A (**36**), B (**37**), D (**38**), M (**39**), N (**40**), and O (**41**)	HEL: 8.7 (**36**), 8.3 (**37**), 16.5 (**38**), 4.9 (**39**), 6.3 (**40**), and 13.4 μM (**41**)	Apoptosis (**39**)
Mantle and glands of *Tritoniopsis elegans* [59]	(1) Extraction in acetone using ultrasound (2) LLE with water and diethyl ether (3) Ether extract subjected to repeated column chromatography (Sephadex LH-20, silica gel, C18 RP HPLC)	Cladiellane-based diterpene family: tritoniopsin B (**42**)	C6: no toxicity HeLa: no toxicity Caco-2: 40–65 μM H9c2: 40–65 μM 3T3-L1: 40–65 μM	-
*Leminda millecra* [64]	(1) Extraction with acetone (2) LLE with ethyl acetate and water (3) Organic phase was purified by silica gel chromatography	Toluhydroquinone: KLM155 (5-methyl-2-[(2′E,6′E)-3′,7′,11′-trimethyl-2′,6′-dodecadien-9′onyl]-1,4-dihydroxybenzene) (**43**)	WHCO1: 9.5 µM WHCO6: 5.8 µM ME180: 33.9 µM SiHa: >150 µM MCF12: 32 µM	Cell cycle arrest and apoptosis; ROS generation; JNK/c-Jun signaling
*Reticulidia fungia* [65]	(1) Extraction in acetone (2) Extraction in dichloromethane (3) Oil residue subjected to repeated column chromatography (silica gel, preparative TLC)	Sesquiterpene carbonimidic dichlorides: reticulidins A (**44**) and B (**45**)	KB: 0.41 (**44**) and 0.42 μg/mL (**45**) L1210: 0.59 (**44**) and 0.11 μg/mL (**45**)	-
*Chromodoris obsoleta* [67]	(1) Extraction with chloroform and methanol (1:3, *v*:*v*) overnight (2) LLE with chloroform and water (3) Chloroform extract subjected to repeated column chromatography (Sephadex LH20, silica gel, RP-HPLC)	Spongian diterpenoids: dorisenone A–D (**46**–**49**); 7α-hydroxyspongian-16-one (**50**); 15α, 16α-diacetoxy-11, 12β-epoxyspongian (**51**); 7α-acetoxydendrillol-3 (**52**); 7α-acetoxy- 17β-hydroxy- 15, 17-oxidospongian- 16-one (**53**); 11β-hydroxyspongi- 12-en- 16- one (**54**); spongian- 16-one (**55**); and 7α-acetoxyspongian- 16-one (**56**)	L1210: 0.21 (**46**), 1.0 (**47**), 7.5 (**48**), 0.8 (**49**), 7.5 (**50**), 0.18 (**51**), 4.8 (**52**), 1.9 (**53**), 1.0 (**54**), 5.0 (**55**), 2.2 μg/mL (**56**) KB: 0.22 (**46**), 1.5 (**47**), 19.0 (**48**), 1.4 (**49**), 10.2 (**50**), 0.98 (**51**), 15.0 (**52**), 2.5 (**53**), 1.9 (**54**), 9.2 (**55**), 16.0 μg/mL (**56**)	-

ER (endoplasmic reticulum); HPLC (high-performance liquid chromatography); LLE (liquid–liquid extraction); ROS (reactive oxygen species); RP (reverse phase); ↑ (increase); ↓ (decrease).

### 3.4. Exemplary Mechanisms of Action

**Figure 8:**
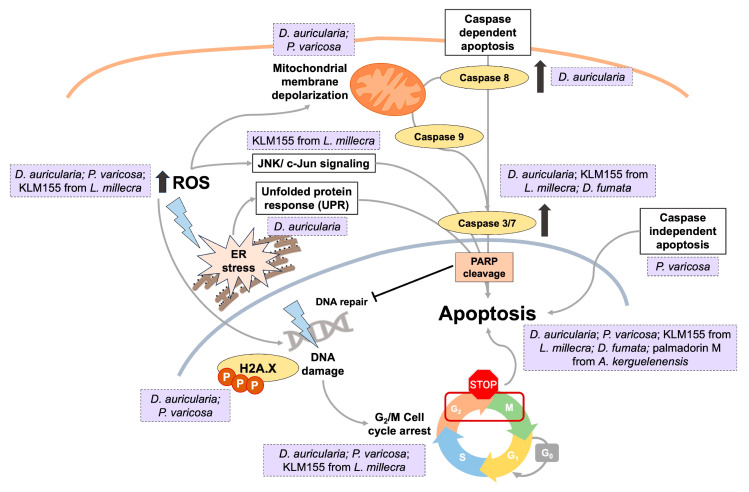
Main mechanisms of action of nudibranch-derived molecules and extracts.

The mechanisms by which compounds and extracts derived from nudibranchs produce their effects are still largely unexplored. However, existing research has identified several mechanisms that contribute to their antitumor activity. These mechanisms include the generation of reactive oxygen species (ROS), induction of DNA damage, arrest of the cell cycle at the G_2_/M phase, and activation of apoptotic pathways [43,46,64,72] (Figure 8).

Huong et al. found that dendrodoristerol (**8**) isolated from *Dendrodoris fumata* exhibited an antitumor effect in HL-60 leukemia cells by inducing apoptosis [46]. This mechanism was evidenced by flow cytometry analysis showing an increased proportion of early apoptotic cells (14.11%), the presence of typical morphological features of apoptosis (16.54%), and the activation of caspase 3 [46]. Similar results were obtained with palmadorin M (**39**) from *Austrodoris kerguelenensis*. This compound has been reported to inhibit Jak2, STAT5, and Erk1/2 activation in HEL cells, leading to the induction of apoptosis. This apoptotic effect was evidenced by apoptosis-specific cleavage of poly-(ADP-ribose) polymerase (PARP) [66].

In addition, extract of methanol–dichloromethane (1:1, *v*:*v*) from the nudibranchs *D. auricularia* and *P. varicosa* demonstrated potent antiproliferative and antitumor effects against CRC cells (HGUE-C-1, HT-29, SW-480), primarily mediated by the induction of cellular stress, DNA damage, and apoptosis. Both extracts promoted the accumulation of intracellular ROS, leading to mitochondrial membrane depolarization, phosphorylation of H2A.X, G_2_/M cell cycle arrest, and subsequent apoptosis. Notably, the extract from *D. auricularia* demonstrated greater potency, particularly through the activation of caspases 3/7 and 8, while the extracts from *P. varicosa* induced caspase-independent apoptosis [43]. In a subsequent study, the *D. auricularia* extract showed selective cytotoxicity against HCT-116 CRC cells, with minimal effects on normal colon fibroblasts (CCD18-Co). Its antitumor activity was mainly mediated through ROS generation, which induced endoplasmic reticulum (ER) stress and activated the unfolded protein response (UPR). This was characterized by ER expansion and upregulation of phospho-JNK, phospho-eIF2α, ATF4, and CHOP proteins. The ROS-mediated ER stress led to DNA damage, G_2_/M arrest, and apoptosis, as evidenced by the activation of caspases 3/7 and 8 and PARP cleavage. Importantly, these cytotoxic effects were significantly reduced in non-tumoral colon cells. Additionally, the *D. auricularia* extract inhibited the proliferation, migration, and invasion of super-invasive HCT-116 populations [72]. Similarly, Whibley et al. reported comparable mechanisms of action for toluhydroquinone KLM155 (**43**), which was isolated from *Leminda millecra*, in the WHCO1 esophageal cancer cell line [64]. The antitumor activity of KLM155 (**43**) was associated with G_2_ phase cell cycle arrest, induction of apoptosis through caspase 3/7 activation, and the generation of ROS. Moreover, activation of MAPK signaling pathways was observed, as evidenced by the increased levels of phosphorylated ERK, p38, and c-Jun. This suggests that JNK/c-Jun signaling plays a key role in mediating these effects in response to cellular stress stimuli [64].

### 3.5. Other Biological Activities

Numerous derivatives from nudibranchs, such as extracts from various *Armina* species as well as fennebricin A (**17**) isolated from *J. funnebris* have potential not only as antitumor agents but also in preventing tumor progression due to their anti-inflammatory properties [77]. Gomes et al. demonstrated the anti-inflammatory activity of extracts from *Armina tigrine*, *A. maculata*, and *A. tricolorata* through a significant reduction in nitric oxide (NO) production in LPS-stimulated RAW264.7 macrophages [73]. This anti-inflammatory activity has been attributed to the presence of n-3 PUFAs, particularly eicosapentaenoic acid (C20:5n-3) and docosahexaenoic acid (C22:6n-3), whose anti-inflammatory properties have been well documented [73,78]. Similarly, the diterpenoid echinoclerodane A isolated from *Hexabranchus sanguineus* has shown a potent inhibitory effect on lipopolysaccharide (LPS)-induced inflammatory responses in RAW264.7, although its antitumor potential remains unexplored [79]. Instead, Huang et al. reported that fennebricin A (**17**) acts as an inhibitor of NF-κB signaling [48]. NF-κB is a crucial transcription factor that regulates immune and inflammatory responses. Its persistent activation contributes to the establishment of a pro-inflammatory tumor microenvironment and is strongly implicated in cancer development and progression by inducing anti-apoptotic genes, promoting uncontrolled cell proliferation, and enhancing angiogenesis, invasion, and metastasis [80,81,82]. Despite some nudibranch derivatives showing pro-oxidative activity, investigating their potential antioxidant properties would also be worthwhile. Marine organisms frequently produce antioxidant compounds as adaptive mechanisms to survive extreme environmental conditions such as high pressure, low temperature, and high salinity as well as oxidative stress-related diseases [83]. While the antioxidant activity of nudibranchs has not yet been studied, potential antioxidant properties have been reported for the sea slug *Elysia crispata*, a similar marine gastropod mollusk belonging to the order *Sacoglossa* [84]. Many natural products exhibit dual redox activity, exhibiting either pro-oxidant or antioxidant effects depending on the cellular context, concentration, and target cell type [85,86,87]. Therefore, exploring the redox-modulating capacity of nudibranch-derived compounds could provide insights into their preventive and antitumor potential.

Nudibranchs have also been recognized for their antimicrobial and antiparasitic potential. For instance, sesterterpenoid compounds demonstrating leishmanicidal activity have been identified from the species *Chromodoris willani* [88]. These compounds, namely deoxymanoalide and deoxysecomanoalide, are believed to be bioconverted by the nudibranch from precursors found in its prey sponge, *Luffariella* sp. Furthermore, compounds with antibacterial activity have also been isolated from *Doriprismatica stellata* nudibranchs and their dietary sponge, *Spongia* cf. *agaricina* [89].

## 4. Discussion and Future Perspectives

Marine organisms are a valuable source of bioactive compounds with promising applications in treating diseases such as cancer. While no compounds derived from nudibranchs have yet advanced to clinical trials or obtained regulatory approval, these organisms have demonstrated significant potential as sources of antitumor agents [10]. Nudibranch derivatives have demonstrated potent cytotoxic activity against various cancer cell lines. However, further studies are needed to clarify the molecular mechanisms of action for the compounds isolated so far, including their specific cellular targets, involved signaling pathways, and potential synergistic effects with existing therapeutic agents. In most cases, they exhibited IC_50_ values lower than those of currently used chemotherapeutic agents, suggesting superior efficacy in vitro [46,48,57,62]. Several marine natural products, including compounds derived from brown algae such as fucoxanthin, fucosterol, and phloroglucinol, have shown potential to enhance the efficacy of chemotherapeutic agents by chemosensitizing cancer cells to 5-fluorouracil [90,91]. Similarly, chrysosporazines derived from marine fungi have demonstrated the ability to reverse P-glycoprotein-mediated resistance to doxorubicin [92,93,94]. Therefore, it would be worthwhile to investigate the synergistic effects of these nudibranch-derived compounds in combination with conventional chemotherapeutics.

Approximately 4700 species of nudibranch have been described worldwide, suggesting a substantial diversity of MNPs associated with these organisms. Expanding the search for bioactive compounds from nudibranchs is particularly relevant, as conventional chemotherapeutic agents often produce severe side effects [8]. To date, dorid nudibranchs have been the most extensively studied concerning their biological potential. In contrast, cladobranch nudibranchs remain relatively underexplored, despite being a promising group due to their distinct diets, which likely lead to different and potentially novel MNP profiles [33]. Moreover, new compounds may still be discovered in previously studied nudibranch species by employing alternative extraction methods or by analyzing various anatomical parts of these organisms.

Finally, it is important to note that bacteria associated with nudibranchs represent another significant source of bioactive molecules [95,96,97]. For example, *Streptomyces* sp. SCSIO 001680, isolated from the Red Sea nudibranch *Chromodoris quadricolor*, exhibited both antimicrobial and antitumor activity [98]. Similarly, compounds produced by *Pseudoalteromonas rubra* (from *Phyllidia coelestis*) and *Virgibacillus salarius* (from *Phyllidia varicosa*) demonstrated activity against methicillin-resistant *Staphylococcus aureus* (MRSA) and cytotoxicity on Vero cells [99]. Consequently, it would be worthwhile to expand the research on nudibranchs to include their microbiome, with the aim of determining the role of these bacteria in the bioactive composition of these organisms.

## 5. Conclusions

Nudibranchs are a group of marine invertebrates that have largely been underexplored regarding their biological and pharmacological potential. This review highlights the remarkable antitumor activity exhibited by a limited number of compounds and extracts derived from nudibranch species studied to date. Various marine natural products (MNPs), primarily hydrophobic in nature, have been extracted from these organisms using organic solvents such as acetone and methanol. Notably, compounds isolated from species such as *Dolabella auricularia*, *Phyllidia varicosa*, and members of the genus *Armina* have demonstrated strong antiproliferative effects against human cancer cell lines, including those of colorectal, lung, and breast origin. Although the precise molecular mechanisms behind these effects are not fully understood, the observed antitumor activity has been mainly associated with the generation of reactive oxygen species (ROS), G_2_/M phase cell cycle arrest, and the induction of apoptosis. Despite these promising findings, substantial research is still necessary to validate the clinical potential of nudibranch-derived compounds. Future efforts should focus on the standardization of extraction methodologies, optimization of compound isolation and purification protocols, comprehensive chemical characterization, and systematic screening of biological activity. Additionally, elucidating the molecular mechanisms of action and performing preclinical in vivo studies will be crucial for advancing these marine natural products toward potential therapeutic applications in oncology, with significant potential to contribute to the development of new cancer treatments.

## Figures and Tables

**Table 1:** Marine natural product-based drugs approved for clinical management of cancer.

Drug Name	Type of Compound	Source Organism	Mechanism of Action	Cancer Treatment Indications
Cytarabine	Nucleoside	*Tectitethya crypta* (sponge)	Cell cycle arrest in S phase by inhibiting DNA synthesis	Acute leukemia
Trabectedin	Alkaloid	*Ecteinascidia* *turbinata* (sea squirt)	DNA alkylating agent, disruption of association of DNA-binding proteins	Ovarian cancer, soft tissue sarcoma, unresectable or metastatic liposarcoma, or leiomyosarcoma
Eribulin mesylate	Macrolide	*Halichondria okadai* (sponge)	Cell cycle arrest in G_2_/M phase by inhibiting microtubule growth	Metastatic breast cancer, unresectable or metastatic liposarcoma
Brentuximab vedotin	Antibody drug conjugate (MMAE)	*Dolabella auricularia* (mollusk)	The antibody targets CD30 and MMAE disrupts microtubule formation	Hodgkin lymphoma

## Data Availability

Not applicable.

## Abbreviations

The following abbreviations are used in this manuscript:

CRC	Colorectal cancer
ER	Endoplasmic reticulum
FDA	Food and Drug Administration
HPLC	High-performance liquid chromatography
LLE	Liquid–liquid extraction
LPS	Lipopolysaccharide
MNPs	Marine natural products
NMR	Nuclear magnetic resonance
NO	Nitric oxide
PUFAs	Polyunsaturated fatty acids
ROS	Reactive oxygen species
RP	Reversed phase
